# Croton Oil and Opium

**Published:** 1845-09

**Authors:** 


*Croton Oil and Opium.*—A writer in the Edinburgh Medical and Surgical Journal, April, 1845, says that 5 drop doses of Croton oil act as a *sedative* cathartic. He treated the African Remittent Fever, with almost uniform success, by the combination of 5 drops of Croton oil with three grains of Opium, after bloodletting and an emetic of Epecacuanha.

He affirms that the same treatment is also very successful in Dysentery. The combination of Croton oil and Opium is declared to be, every way, preferable to Calomel.

As soon as a free evacuation of the bowels is induced, the Sulphate of Quinine is exhibited liberally, to prevent a return of paroxysm.—*Western Lancet.*

